# Exploratory factor analysis of constructs used for investigating research uptake for public healthcare practice and policy in a resource-limited setting, South Africa

**DOI:** 10.1186/s12913-023-10165-8

**Published:** 2023-12-15

**Authors:** Jerry Sigudla, Jeanette E. Maritz

**Affiliations:** Department of Health Studies, University of South Africa, Pretoria, South Africa

**Keywords:** Healthcare policy, Healthcare practice, Low-resource settings, Uptake of public health research

## Abstract

**Background:**

Low-resource settings are often less capable of responding to and implementing available quality research evidence for public healthcare practice and policy development due to various factors. In most low-resource settings, limited empirical evidence is available to help deal with localised factors that contribute to low public health research uptake, particularly from the perspective of key research stakeholders.

**Methods:**

Although the study initially employed a two-phase exploratory sequential approach, this paper focuses on the results generated from a quantitative approach. Considering the determining factors that affect research uptake in the context of low-resource settings, a measuring instrument was developed and its reliability and validity were assessed using an exploratory factor analysis approach.

**Results:**

A total of 212 respondents, according to their job roles and titles, were identified as researchers, front-line workers, programme managers, and directors/senior managers of higher learning institutions, indicating that the three constructs applied in the questionnaire, namely (1) individual factors, (2) organisational factors, and (3) research characteristics, demonstrated relatively high reliability with a Cronbach’s alpha of greater than 0.791.

**Conclusion:**

The study concludes that the instrument can potentially be used to measure factors that affect research uptake in low-resource settings.

**Supplementary Information:**

The online version contains supplementary material available at 10.1186/s12913-023-10165-8.

## Background

It is widely known that in low-resource countries, the use of health research for practice and policy development is very low, and more research is required to develop strategies that could improve the adoption of research [1, 2]. Although the concept of research uptake has received attention in these settings, efforts to promote research uptake into healthcare practice and policy development remain hindered by several competing priorities [3]. This requires low-resource countries to remain innovative to ensure that high-quality research studies are successfully implemented. Several scholars have suggested that most research uptake challenges could be mitigated by developing customised and impactful strategies that are suitable for the local context [4].

The concept of research uptake advocates for proactive collaboration between researchers and all relevant stakeholders. Research uptake is defined as a process by which knowledge generated through research enters the domain of audiences such as practitioners, scholars, end users, policy makers in government and other agencies [5]. However, these engagements start with the inception of a research project. The Development Research Uptake in Sub-Saharan Africa (DRUSSA) emphasises that research uptake is a comprehensive process that focuses on the entire research cycle, from proposal right through practice and policy development [6]. In the past, the relationship between health researchers and end users has historically varied; in some cases, limited participation has negatively affected the acceptance of health research for practice and policy [7].

Consequently, this situation of low research uptake demands a more fine-grained understanding of factors affecting research uptake in resource constraints settings, so that targeted innovative strategies are developed and put in place to improve research uptake. However, there remains a lack of adequate models for active facilitation of research uptake in low-resource countries [8]. This situation limits the amount of knowledge on specific factors affecting research uptake in developing countries compared to developed countries. The lack of quantitative evidence for such models also limits the ability to understand and stimulate a way of thinking about promoting research uptake in low-resource settings [2]. Therefore, the development of instruments for measuring research uptake is vital to address existing knowledge gaps in this particular area. Furthermore, the instrument will help establish credible management tools to measure public health research uptake initiatives and performance. This paper aims to contribute to filling this important evidence gap by identifying and providing empirical support for the reliability of the instrument developed to determine factors associated with research uptake in a rural setting in South Africa.

## Development of the Survey Questionnaire

From the qualitative phase of this study, a pool of items was generated that were eligible for inclusion in the data collection instrument. In consultation with the co-author and guided by the literature, the researcher finalised the instrument to address local issues as raised during the in-depth interviews. The questions were selected in order of relevance and used to determine the factors affecting research uptake in the local context. In the current study, a 5-point Likert scale-style survey questionnaire was developed and used to collect online data from respondents [9].

A pilot study was conducted to pre-test the questionnaire by selecting 20 respondents who, because of their job roles and titles, were identified as researchers, frontline workers, programme managers and directors of institutions of higher learning. Based on feedback received from the respondents, the instrument was finalised with improvements and modifications in terms of spelling, language structure, and clarity. As a result, a complete questionnaire was developed, which comprised 61 Likert scale questions / items to determine the factors that affect research uptake.

## Methods

### Study design

Although the study initially employs a two-phase exploratory sequential approach, this paper focuses on the results generated from a cross-sectional quantitative approach (see Additional file 1: STROBE checklist).

### Study setting and population

The study was carried out in Mpumalanga Province, a rural province in South Africa with just over 4.7 million people, representing 7.8% of the total population of the country [10]. According to data from internal records (research files) of our province, 399 public health research studies were conducted between 2014 and 2019. This translates into an equivalent of 67 public health research studies conducted per year. Therefore, the researcher emailed survey questionnaires to all identified research stakeholders who conducted research in the province from 2014 to 2019.

Of the 399 internal public health research studies registered, a total of 187 were excluded for the following reasons: refusal to participate (n = 26); participation in the qualitative phase (n = 21); email bounced and was untraceable (n = 30); and email went through, but respondents chose not to participate despite two reminders (n = 110). This resulted in a response rate of approximately 53%. The data for this analysis were derived from a total of 212 respondents who, according to their job roles and titles, were identified as researchers, front-line workers, programme managers, and directors or senior managers of institutions of higher learning.

### Data analysis

Considering that this research area is new in the current setting, the elements were tested using an exploratory factor analysis (EFA) with the aim of measuring the internal consistency of the elements and determining the number of factors and elements for each construct. EFA is a technique that statistically explores the underlying factors of a variable through factor loading values so that researchers assume that some indicators may be related to several factors [11]. There are several types of EFA, including (a) common factor analysis (CFA), used when a primary objective of the research looks at how well a new set of data fits a model; (b) principal component analysis (PCA), mainly used to identify the factor structure for a set of variables; and (c) confirmatory factor analysis (CFA), which is based on a strong theoretical foundation that allows the researcher to specify an exact model in advance [12]. In this paper, principal component analysis (PCA) as a factor extraction method is of primary interest. Furthermore, the internal consistency of the elements under each component of the research uptake constructs was analysed using Cronbach’s alpha (alpha coefficient), which is defined as a measure of the reliability of the responses of the data collection instrument [11, 12].

## Results

The researchers established a total of 61 Likert scale statements/items from the survey responses as contributory factors that affect research uptake. Of these, 21 statements/items were used to measure individual factors, 20 items to measure organisational factors, and 20 items to measure research characteristics.

### Sample adequacy and data suitability for factor analysis

Initially, the factorability of the 61 Likert scale statements/items was examined using the Kaiser‒Meyer‒Olkin (KMO) test and Bartlett’s test of sphericity (see Table 1) [12]. The KMO test is used to determine the suitability of data for factor analysis. In other words, it tests whether there is enough strong factor structure. The KMO test values range from 0 to 1. Values between 0.8 and 1.0 indicate that the sampling is adequate, values between 0.7 and 0.79 indicate moderate sampling, and values between 0.6 and 0.69 indicate mediocre sampling. KMO values less than 0.6 indicate that the sampling is not adequate for factor analysis [12, 13]. Bartlett’s test of sphericity tests the hypothesis that the correlation matrix is an identity matrix, which would indicate that the variables are unrelated and, therefore, unsuitable for structure detection. In other words, it determines whether there are significant correlations between any of the different items. A significant value of < 0.05 indicates that a factor analysis may be worthwhile for the data set.

**Table 1:** Result of factor analysis for all constructs

Construct	Kaiser‒Meyer‒Olkin Measure of Sampling Adequacy.	Bartlett’s Test of Sphericity Significant Value Result
Individual factors	0.883	0.000
Organisational factors	0.841	0.000
Research Characteristics	0.791	0.000

The results in Table 1 indicate that Bartlett’s test of sphericity is significant for all constructs, with a p-value of < 0.05. The KMO sampling adequacy measure for all constructs indicated values close to 1.0, which exceeded the recommended threshold value of above 0.6.

### Exploratory factor analysis for individual factors

The KMO of 0.883 is a strong enough factor structure above 0.6, and the Bartlett test of sphericity is significant (p < 0.05). This suggests that there are significant correlations.

#### Number of factors affecting research uptake for the construct: individual

**Fig. 1:**
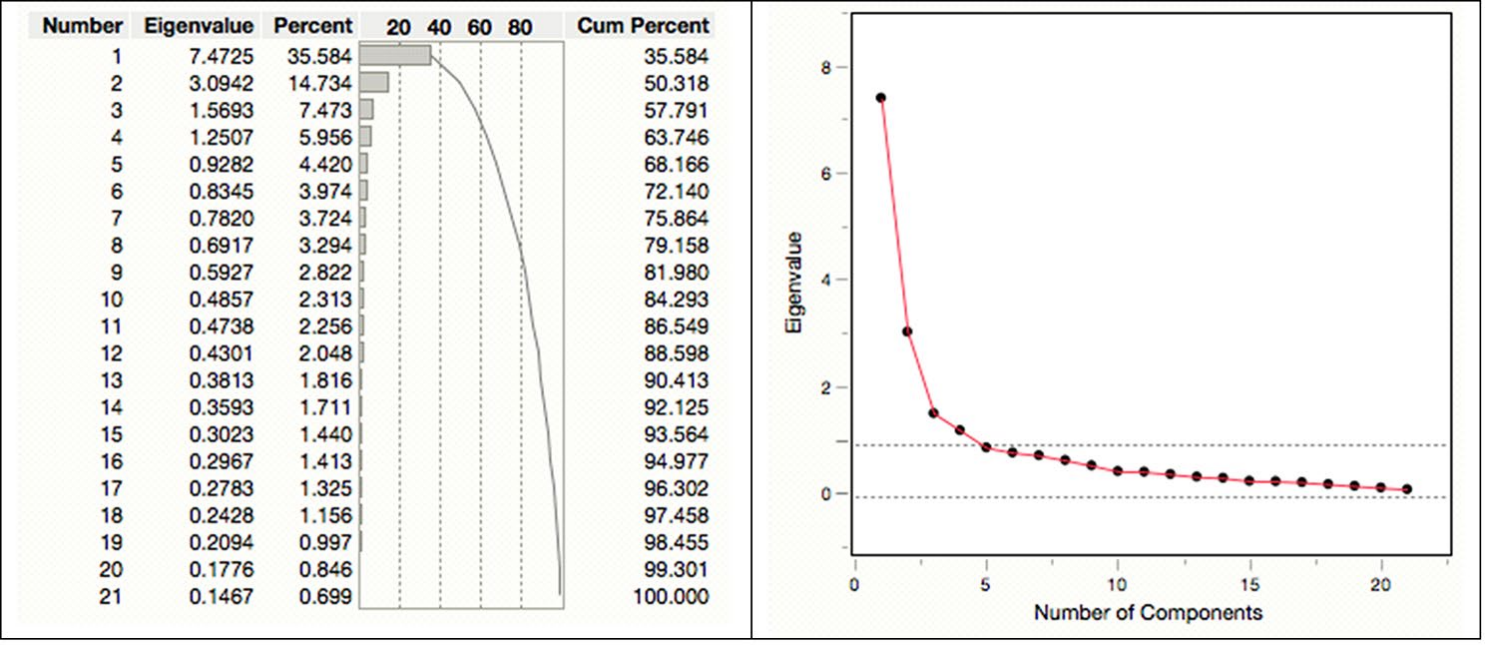
The total variance plot indicating the eigenvalues for individual factors, and the scree plot

In deciding on the number of factors, the total variance plot with criteria of eigenvalues greater than 1.0, scree plot and cumulative percentage of variance above 60% were used [11]. The principal component analysis for the construct: *individual factors*, extracted four components with eigenvalues greater than 1.0 (see Fig. 1). The plot shows four possible factors that explain 63.75% of the variance, which is above the acceptable threshold of 60%. This means that, in terms of the cumulative sums extracted from the loading value, the four factors extracted can explain 64% of the construct.

The scree plot confirms that the first four factors for the construct account for most of the data’s total variability, with the remaining factors accounting for the remaining small proportion of the variability, and is therefore, less important. For this construct, the solution is the choice of four factors.

#### Rotated factor loading for individual factors

Principal component analysis with varimax rotation was used to obtain the factor load. This procedure was important in determining which items belong to which factor and was used as a tool for item reduction. In interpreting the rotated factor pattern, an item was said to be loaded onto a given factor if the factor loading was 0.40 or greater for that factor and less than 0.40 for the other factors [14]. The 21 Likert scale statements/items were designed to index four factors, namely *support*, *experience*, *motivation*, and *time constraints*. This is shown in Table 2 below, with loadings less than 0.40 dimmed to improve clarity.

**Table 2:** Matrix of rotated factor loadings (individual factors)

Item	Factor 1	Factor 2	Factor 3	Factor 4
C16	**0.93**	0.04	-0.1	-0.1
C14	**0.84**	-0.0	-0.0	-0.0
C19	**0.75**	-0.0	0.10	-0.0
C15	**0.70**	-0.1	-0.1	0.08
C10	**0.57**	0.09	-0.0	0.29
C20	**0.50**	-0.0	0.14	0.06
C21	0.30	0.17	0.17	0.14
C4	0.00	**0.78**	-0.0	0.05
C2	-0.0	**0.75**	0.03	-0.1
C3	-0.1	**0.67**	0.02	0.07
C5	-0.1	**0.63**	0.14	0.12
C1	0.28	**0.61**	0.01	-0.1
C12	-0.0	-0.1	**0.82**	0.07
C13	0.00	-0.0	**0.80**	-0.0
C11	0.08	-0.0	**0.71**	0.03
C18	0.04	0.17	**0.60**	-0.0
C17	-0.0	0.19	**0.39**	-0.0
C9	0.04	-0.0	0.06	**0.79**
C8	-0.1	0.00	0.02	**0.77**
C7	0.29	0.10	-0.0	**0.62**
C6	0.39	0.19	-0.1	**0.48**

Suppress absolute loading value less than 0.3, Dim Text0.4

The table above shows the factor loading of 21 items under four factors. In this case, Factor 1 had strong positive loadings on the first six items (C16, C14, C19, C15, C10, and C20), and it is named *support*. The subsequent five items (C4, C2, C3, C5, and C1) had high loadings and belonged to Factor 2, which is named *experience*. The third factor, named *motivation*, loaded highly on the next four items in the table (items C12, C13, C11, and C18), with item C17 indexed low on motivation, although still positive. The last factor, *time constraints*, had strong loadings for items C9, C8, and C7, and a positive low loading (0.48) for item C6. There was less cross-loading from all factors, with all loadings greater than 0.4. Due to the low factor loading measured, only one item (C21) was not loaded from the original 21 items.

#### Reliability analysis for the scale of individual factors

The Cronbach’s alpha coefficient was used to calculate the internal consistency or reliability of a set of elements. The criteria for the interpretation of the Cronbach’s alpha coefficient for reliability are that any value above 0.8 is a good reliability, between 0.6 and 0.8 is an acceptable reliability, and a Cronbach’s alpha coefficient value below 0.6 is an unacceptable reliability for exploratory research [14]. Additional file 2: Table S1 shows a test for the reliability of the construct: *individual factors*.

The Cronbach’s alpha values are 0.8853, 0.8385, 0.8323, and 0.8668 for Factors 1 to 4 respectively, indicating good reliability. This implies that the individual elements of the construct measured the same construct consistently. Furthermore, the reliability measure for the four consolidated factors also exceeds the minimum value of 0.6, with a value of 0.901.

### Exploratory factor analysis for organisational factors

The KMO of 0.841 is strong enough for a factor structure greater than 0.6, and Bartlett’s sphericity test is significant (p < 0.05). This suggests that there are significant correlations.

#### Number of factors affecting research uptake for the construct: Organisational

PCA for the construct: *organisational factors* extracted four components with eigenvalues exceeding 1.0. As indicated in the figure below, the selected four factors have rotation sum squared loadings equal to 70.55%, which is above the acceptable threshold of 60%. This indicates that approximately 71% of the total variance is explained by these four factors of the construct.

**Fig. 2:**
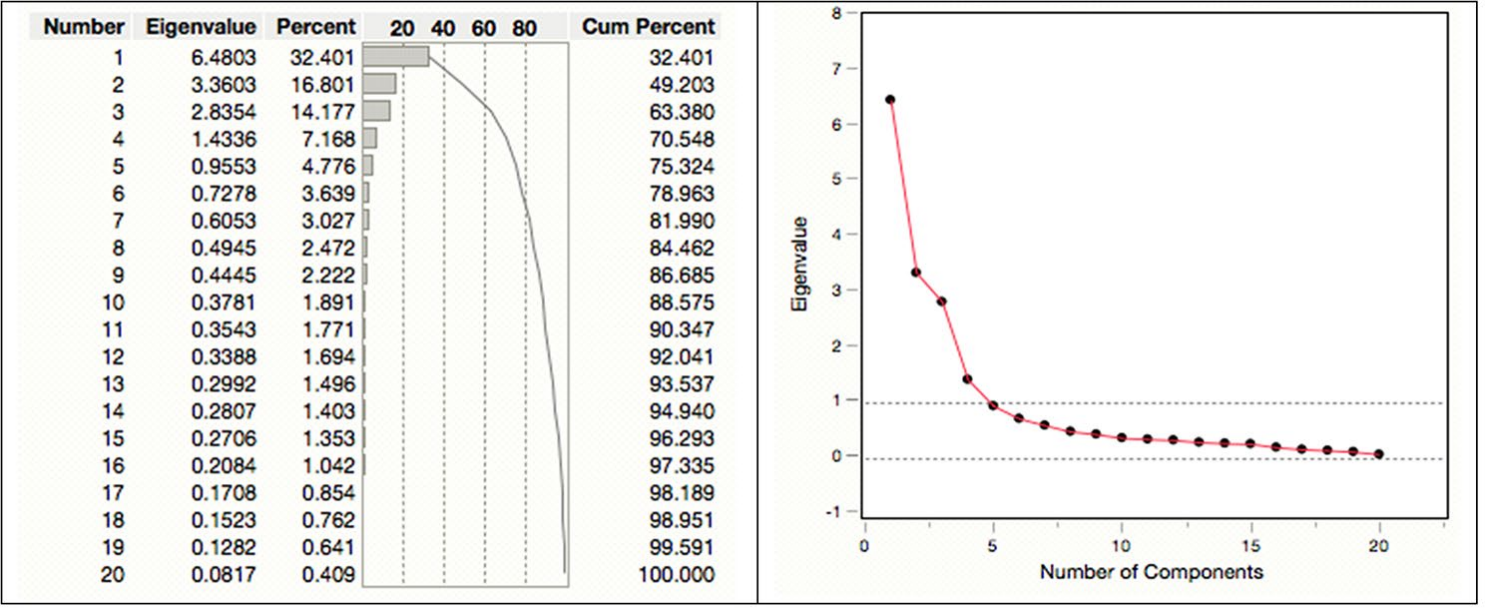
The total variance plot indicating eigenvalues for organisational factors, and the scree plot

Similarly, the scree plot obtained from the output results on the construct is shown in Fig. 2. The plot further confirms that the first four factors account for most of the total variability in the data and are, indeed, the largest. The remaining factors are probably unimportant, as they account for a very small proportion of the variability. For this construct, the solution is the choice of four factors.

#### Rotated factor loading for organisational factors

PCA with varimax rotation was used to obtain the factor load, and the 20 Likert scale statements/items were indexed into four factors, namely *resources*, *partnerships*, *research agenda*, and *private funders*. The rotated component matrix for organisational factors affecting research uptake is shown in the table below, with loadings less than 0.40 dimmed to improve clarity.

**Table 3:** Matrix of rotated factor loadings (organisational factors)

Item	Factor 1	Factor 2	Factor 3	Factor 4
**D2**	**0.90**	0.01	0.01	0.00
**D1**	**0.89**	0.06	-0.0	-0.0
**D3**	**0.87**	-0.1	0.10	0.05
**D4**	**0.81**	0.00	0.07	-0.0
**D5**	**0.40**	0.03	0.03	0.03
**D13**	0.05	**0.87**	-0.1	-0.0
**D12**	0.04	**0.86**	-0.0	-0.1
**D14**	-0.0	**0.85**	-0.0	0.04
**D15**	-0.1	**0.72**	0.07	-0.0
**D11**	0.03	**0.61**	0.23	0.04
**D7**	0.03	0.04	**0.86**	-0.1
**D9**	0.06	-0.0	**0.82**	-0.0
**D6**	0.01	0.03	**0.79**	-0.0
**D8**	0.11	0.02	**0.75**	-0.0
**D10**	-0.0	0.13	**0.55**	0.10
**D18**	0.07	-0.1	0.00	**0.87**
**D17**	0.15	0.02	-0.1	**0.87**
**D19**	0.10	-0.0	-0.1	**0.78**
**D16**	0.03	0.12	-0.1	**0.72**
**D20**	-0.2	-0.0	0.17	**0.55**

Suppress absolute loading value less than 0.3, Dim Text0.4

The results from Table 3 show that the first factor had the highest positive loadings on the first five indicated items (D2, D1, D3, D4, and D5), and it is named resources. Similarly, the second factor had strong loadings on the next five items (D13, D12, D14, D15, and D11) and accordingly named partnerships. The third factor loaded strongly on the subsequent five items in the table (items D7, D9, D6, D8, and D10), and it was named the research agenda. The last factor, named *private funders*, had high loadings for items D18, D17, D19, D16, and D20. There was no cross-loading from all factors, as illustrated in the table above.

#### Reliability analysis for the scale of organisational factors

Additional file 3: Table S2 shows a test of the reliability coefficient values of the final elements in this study using Cronbach’s alpha, for the construct: *organisational factors*.

The values of all Cronbach’s alphas are 0.8868, 0.8669, 0.8874, and 0.9028 for components 1 to 4, respectively, which indicate good reliability. This implies that the 20 Likert scale statements/items of the construct measured the same construct consistently. Furthermore, the overall reliability measure for the four consolidated components (i.e., 20 items) also exceeds the minimum value of 0.6, with a value of 0.878, and is therefore deemed reliable.

### Exploratory factor analysis for research characteristics

The KMO of 0.791 is a strong enough factor structure above 0.6, and the Bartlett’s test of sphericity is significant (p < 0.05). This suggests that there are significant correlations.

#### Number of factors affecting research uptake of the construct: research characteristics

The total variance explained by these generated factors is shown in the figure below on the research characteristics factors affecting research uptake. There are five factors with variances (eigenvalues) that are greater than 1. As illustrated in the table, the five selected factors have rotation sum squared loadings equal to 65.20%, which is above the acceptable threshold of 60%. This indicates that approximately 65% of the total variance is explained by these five factors.

**Fig. 3:**
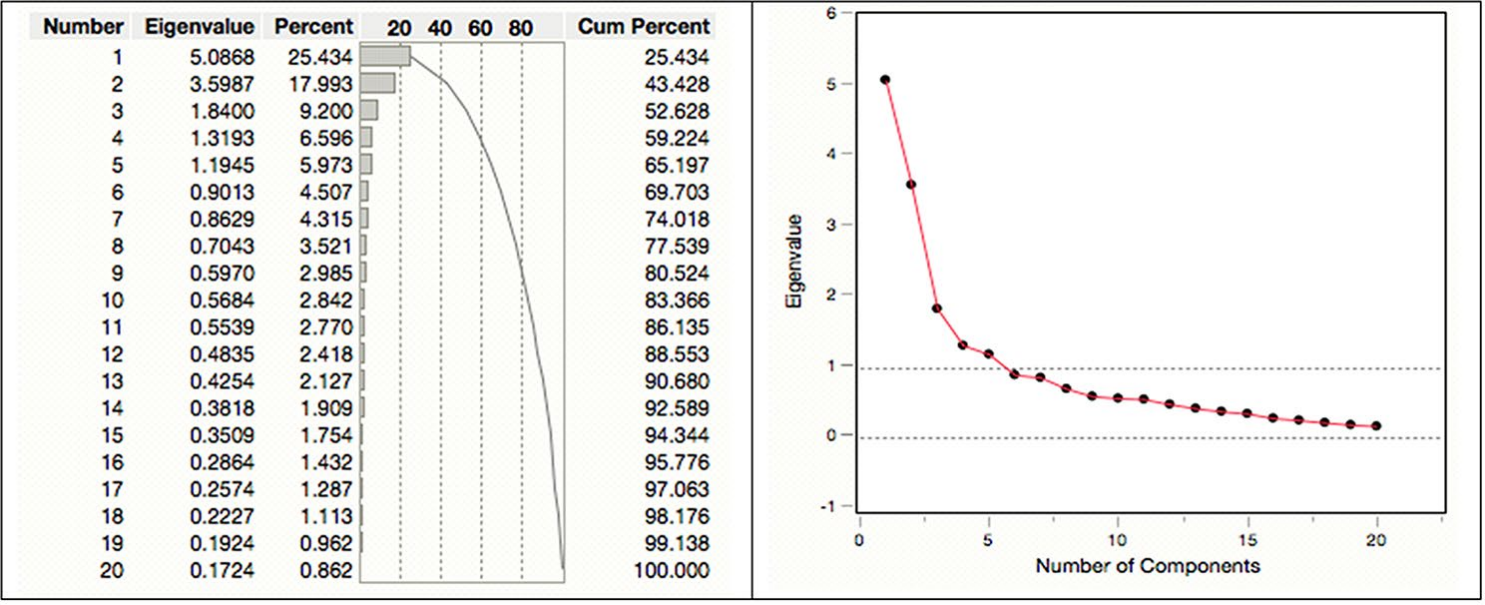
The total variance plot indicating eigenvalues for research characteristics, and a scree plot

The scree plot (Fig. 3) obtained from the output results confirms the selection of the five factors that account for most of the total variability in the data. The remaining factors are likely unimportant, as they account for a very small proportion of the variability. For this construct, the choice of five factors appear to be the solution.

#### Rotated factor loading for research characteristics factors

Similarly, PCA with varimax rotation was conducted to assess the five ‘research characteristics’ variables clustered. These variables are indexed into five factors and are named *gate-keeping process*, *local research committees*, *accessibility of evidence*, *quality of evidence*, and *critical appraisal skills*. The rotated component matrix for research characteristics factors affecting research uptake is shown in Table 4 below, with loadings less than 0.40 dimmed to improve clarity.

**Table 4:** Matrix of rotated factor loadings (research characteristics factors)

Item	Factor 1	Factor 2	Factor 3	Factor 4	Factor 5
**E13**	**0.83**	-0.1	-0.0	0.09	-0.1
**E11**	**0.82**	-0.0	-0.1	0.13	-0.0
**E14**	**0.81**	0.01	0.12	-0.1	0.09
**E12**	**0.80**	0.02	-0.0	-0.1	0.05
**E15**	**0.73**	0.09	0.09	-0.0	0.08
**E16**	**0.54**	0.20	-0.1	-0.0	-0.1
**E19**	-0.0	**0.83**	-0.0	0.10	0.00
**E18**	0.02	**0.81**	0.08	-0.1	0.03
**E20**	0.04	**0.73**	-0.1	0.01	0.06
**E17**	***0.42***	***0.48***	*0.05*	*0.04*	*-0.1*
**E1**	-0.1	0.15	**0.62**	0.16	-0.1
**E2**	0.04	-0.1	**0.58**	0.06	0.07
**E4**	-0.0	0.00	**0.57**	-0.1	0.04
**E5**	0.04	-0.2	**0.53**	0.17	-0.0
**E3**	0.02	0.07	0.31	0.16	0.19
**E7**	0.02	0.03	0.12	**0.74**	0.03
**E6**	-0.1	0.03	0.10	**0.69**	0.01
**E8**	0.06	-0.0	-0.0	**0.42**	0.39
**E9**	-0.0	0.01	-0.1	0.06	**0.63**
**E10**	0.03	0.06	0.19	-0.1	**0.62**

Suppress absolute loading value less than 0.3, Dim Text0.4

The first factor, which is named the *gatekeeping process*, had strong loads on seven items (E13, E11, E14, E12, E15, and E16). There was cross-loading on Item E17 for Factor 1 and Factor 2, and therefore, this item (*the local research committee is ensuring that the research conducted is geared toward improving service delivery*) is omitted as a contributory item in either of the factors. The second factor had high loadings on the next three items (E19, E18, and E20), and it was named *local research committees*. Factor 3 is named *accessibility of evidence* and loaded positively high on the subsequent four items in the table (items E1, E2, E4 & E5). The fourth factor had strong loadings for items E7, E6, and E8 and was named *quality of evidence*. There was a fifth factor named *critical appraisal skills*, which loaded strongly positive on two items (E9 & E10). However, because there are only two items that depict Factor 5, more evidence is required to associate the items with the Factor [14]. Item E3 (*there is a lack of research evidence relevant to my work context*) is also omitted, as it has a loading of 0.31.

#### Reliability analysis for the construct: research characteristics

All Cronbach’s alpha values demonstrate satisfactory internal consistency reliability of all dimensions. The Cronbach’s alpha values are 0.8915, 0.8442, 0.6914, 0.7367, and 0.6546 for Factors 1 to 5, respectively, and exceed the acceptable minimum threshold of 0.6 (see Additional file 4: Table S3).

Furthermore, the reliability measure for the five consolidated components (i.e., 18 items) also exceeds the minimum value of 0.6, with a value of 0.791, which is an acceptable reliability.

## Discussion

The main objective of this paper was to provide empirical support for the reliability of the instrument developed to determine factors associated with research uptake in a rural setting in South Africa. This was conducted through an item-level analysis using exploratory factor analysis. The results reveal that the questionnaire employed to measure all three constructs (i.e., individual factors, organisational factors, and research characteristics) is reliable with a high Cronbach’s alpha value of above 0.6 [14]. The factor analysis produced four factors for individual factors, four factors for organisational factors, and five factors for research characteristics. The principal component analysis revealed that each component was able to explain between 64% and 71% of the construct, which is above the acceptable threshold of 60% [11]. The results of the reliability analysis show that each item in the three constructs contributes to the overall reliability of the instrument at a high Cronbach’s alpha value, within the range of 0.791 and 0.901. Thus, the results explicitly demonstrate that the items proposed in this instrument are relevant in this research setting and in the context of low-resource settings.

The results of factor analysis show that individual factors were measured using four significant and meaningful constructs of research uptake. These four constructs (19 items) of individual factors are categorised as *support*, *experience*, *motivation*, and *time constraints*. Similarly, the structure of organisational factors was measured using four significant and meaningful constructs (20 items) of research acceptance: *resources*, *partnerships*, *research agenda*, and *private funders*. However, the research characteristics appeared to have five meaningful constructs (18 items) of research uptake. These five constructs are categorised as *gate keeping process*, *local research committees*, *accessibility of evidence*, *quality of evidence*, and *critical appraisal skills*. These results were key in the identification of the suitable factors affecting research uptake and, as a result, a total of four low factor loading items were identified and eliminated from the analysis because it was assumed that they were not suitable for the study context. Although the Cronbach’s alpha value for each construct exceeded the minimum 0.50 threshold as proposed by scholars [15], more analyses are needed to confirm reliability and validity measurements.

### Study limitations

Although the research was conducted satisfactorily, certain limitations should be considered. These limitations are discussed below and are expected to be addressed in future research. First, the research team felt that several stakeholders were left out in this study, which is a limitation. These included the public (community members/patients), politicians, and healthcare managers without practical research experience. Their inclusion in this study could have added another dimension to looking at the uptake of research. While the inclusion of different categories of stakeholders from various organisations / institutions is a strength, the study was conducted in a rural province of South Africa using convenience sampling. Therefore, it cannot be assumed that the findings represent all situations in low-resource countries, as settings could be unique.

## Conclusion

The authors in the current study recommend the use of this instrument for confirmatory factor analysis in settings similar to those of the current study. The availability of a valid and reliable instrument is essential to guarantee validity and reliability and provide credible alternative instruments for measuring research uptake initiatives for public healthcare practice and policy development.

### Electronic supplementary material

Below is the link to the electronic supplementary material.

Supplementary Material 1

Supplementary Material 2

Supplementary Material 3

Supplementary Material 4

## Data Availability

Additional data that support these findings are available from the author (Dr J Sigudla: sigudla@yahoo.com) upon reasonable request.

## Abbreviations

EFA: Exploratory Factor Analysis
KMO: Kaiser‒Meyer‒Olkin test
PCA: Principal component analysis
