# Supplementary material for: Exploratory factor analysis of constructs used for investigating research uptake for public healthcare practice and policy in a resource-limited setting, South Africa
Source: BMC Health Serv Res. 2023 Dec 15;23:1423. doi: 10.1186/s12913-023-10165-8 (PMC10724913; doi:10.1186/s12913-023-10165-8)
Supplement: Supplementary file 2 — Supplementary Material 2 [file 12913_2023_10165_MOESM2_ESM.docx]

Table S1: Reliability analysis of the scale for individual factors

| **Factors** | **Question Code** | **Statement** | **Cronbach’s Alpha (α)** | **Interpretation** |
| --- | --- | --- | --- | --- |
| **Support (CF1)** | C10 | My organisation gives me a protected time to do research. | 0.8853 | Good |
|  | C14 | There is proper research mentoring in my organisation. |  |  |
|  | C15 | There are financial incentives to promote the uptake of research. |  |  |
|  | C16 | There is broad support within the organisation at all levels on research-related matters. |  |  |
|  | C19 | I believe that research is valued by my colleagues. |  |  |
|  | C20 | I believe research is valued by government. |  |  |
| **Experience (CF2)** | C1 | I have adequate exposure to research methods. | 0.8385 | Good |
|  | C2 | I have a clear understanding of research methods. |  |  |
|  | C3 | I have adequate experience in putting research evidence into practice. |  |  |
|  | C4 | I have sufficient knowledge to search the literature to retrieve research evidence. |  |  |
|  | C5 | I can determine the applicability of the research findings. |  |  |
| **Motivation (CF3)** | C11 | I am always motivated by the desire to promote the use of research for practice. | 0.8323 | Good |
|  | C12 | I am always motivated by the desire to come up with creative ideas to improve something. |  |  |
|  | C13 | I am always motivated by the desire to learn new things. |  |  |
|  | C18 | I have the responsibility to keep up to date with the latest research evidence. |  |  |
| **Time factor (CF4)** | C6 | I have enough time in my office to look up research articles/reports. | 0.8668 | Good |
|  | C7 | My workload allows me to keep up to date with all the latest research evidence. |  |  |
|  | C8 | I have enough time at home to search for articles / reports. |  |  |
|  | C9 | My personal responsibilities allow me to stay up-to-date with new research evidence. |  |  |
| The overall Cronbach's alpha for all factors | | | 0.901 | Good |
