# Supplementary material for: Exploratory factor analysis of constructs used for investigating research uptake for public healthcare practice and policy in a resource-limited setting, South Africa
Source: BMC Health Serv Res. 2023 Dec 15;23:1423. doi: 10.1186/s12913-023-10165-8 (PMC10724913; doi:10.1186/s12913-023-10165-8)
Supplement: Supplementary file 3 — Supplementary Material 3 [file 12913_2023_10165_MOESM3_ESM.docx]

Table S2: Reliability analysis of the scale for organisational factors

| **Factors** | **Question code** | **Statement** | **Cronbach’s Alpha (α)** | **Interpretation** |
| --- | --- | --- | --- | --- |
| **Local Research Agenda (DF3)** | D6 | The government has a clear research agenda. | 0.8868 | Good |
|  | D7 | The government's research agenda has been clearly communicated. |  |  |
|  | D8 | The research agenda is current and addresses real-life problems that affect government. |  |  |
|  | D9 | I have an adequate understanding of the government's research agenda. |  |  |
|  | D10 | Most research studies conducted are based on the government's research agenda. |  |  |
| **Funding (DF4)** | D16 | Private funders of research play a significant role in promoting research uptake. | 0.8669 | Good |
|  | D17 | Private funders of research assist in building local capacity through research projects. |  |  |
|  | D18 | Private funders of research drive performance and improve standards in government institutions. |  |  |
|  | D19 | Private funders of research play a critical role in research on community stakeholder involvement. |  |  |
|  | D20 | Key research questions chosen by private funders of research is always aligned with the research agenda of decision makers. |  |  |
| **Resources (DF1)** | D1 | Research is sufficiently prioritised by my organisation. | 0.8874 | Good |
|  | D2 | My organisation invests substantial resources to improve research capacity. |  |  |
|  | D3 | My organisation has enough manpower to support research activities. |  |  |
|  | D4 | My organisation has sufficient resources available to influence research uptake. |  |  |
|  | D5 | My organisation is selective on which researchable condition it focuses on. |  |  |
| **Partnerships (DF2)** | D11 | The government effectively collaborates with other research institutions to promote research use. | 0.9028 | Good |
|  | D12 | The government frequently engages with researchers to find researched solutions. |  |  |
|  | D13 | There is active engagement from government with stakeholders at all stages of the research being conducted. |  |  |
|  | D14 | There is proper communication between the government and various groups involved in research matters. |  |  |
|  | D15 | Government has platforms for stakeholders with related interests to engage in research matters. |  |  |
| The overall Cronbach’s alpha for organisational factors | | | 0.878 | Good |
