# Supplementary material for: Exploratory factor analysis of constructs used for investigating research uptake for public healthcare practice and policy in a resource-limited setting, South Africa
Source: BMC Health Serv Res. 2023 Dec 15;23:1423. doi: 10.1186/s12913-023-10165-8 (PMC10724913; doi:10.1186/s12913-023-10165-8)
Supplement: Supplementary file 4 — Supplementary Material 4 [file 12913_2023_10165_MOESM4_ESM.docx]

Table S3: Reliability analysis of the scale for research characteristics factors

| **Factors** | **Question code** | **Statement** | **Cronbach’s Alpha (α)** | **Interpretation** |
| --- | --- | --- | --- | --- |
| **Gate keeping process (EF1)** | E11 | The government has a clear approval process for granting permission to conduct research. | 0.8915 | Good |
|  | E12 | The government's approval process for permission to conduct research has been clearly communicated. |  |  |
|  | E13 | I have a clear understanding of the government approval process for permission to conduct research. |  |  |
|  | E14 | The government approval process for permission to conduct research is short and easy to carry out. |  |  |
|  | E15 | Feedback on the government approval process for permission to conduct research is communicated in a timely way. |  |  |
|  | E16 | I am fully aware of the role of the Provincial Health Research Committee in facilitating the uptake of research. |  |  |
| **Local Research Committees (EF2)** | E18 | The Provincial Health Research Committee is ensuring that research findings are channelled to decision makers. | 0.8442 | Good |
|  | E19 | The Provincial Health Research Committee is actively involved in all stages of the research being conducted. |  |  |
|  | E20 | The Provincial Health Research Committee ensures that research results are always communicated back to the department by researchers. |  |  |
| **Accessibility of evidence (EF3)** | E1 | There is poor access to relevant, high-quality research. | 0.6914 | Acceptable |
|  | E2 | There is a lack of delivery of research results to target audiences. |  |  |
|  | E4 | There is a lack of resources (web-based) to access research evidence within government. |  |  |
|  | E5 | There is a lack of communication between researchers and decision makers for the dissemination of research findings. |  |  |
| **Quality of evidence (EF4)** | E6 | Most of the research evidence is of poor quality. | 0.7367 | Acceptable |
|  | E7 | Presentation of research evidence not detailed enough for decision-making. |  |  |
|  | E8 | Most research articles are not relevant to my work activities. |  |  |
| **Critical appraisal skills (EF5)** | E9 | Research articles are difficult to understand due to research jargon. | 0.6546 | Acceptable |
|  | E10 | I have difficulty judging the quality of research findings in articles and reports. |  |  |
| The overall Cronbach's alpha for research characteristics factors | | | 0.791 | Acceptable |
